# Teaching the electronic health record in a technical nursing program

**DOI:** 10.1590/0034-7167-2025-0641

**Published:** 2026-10-16

**Authors:** Renata da Paixão Pereira, Christine Barbosa Betty, Nildo Alves Batista

**Affiliations:** I Instituto de Ensino e Pesquisa da Santa Casa de São José dos Campos. São José dos Campos, SP, Brazil; II Universidade Federal de São Paulo. São Paulo, SP, Brazil

**Keywords:** Education, Nursing, Associate, Health Literacy, Electronic Health Records, Technology and Innovation Policy, Teaching., Graduación en Auxiliar de Enfermería, Alfabetización en Salud, Registros Electrónicos de Salud, Tecnología e Innovación, Enseñanza.

## Abstract

**Objective:**

To analyze teaching and learning related to the electronic health record (EHR) in a technical nursing program, identifying weaknesses and strengths to inform improvements to the program’s pedagogical project.

**Method:**

This qualitative, exploratory, descriptive study used document analysis and fieldwork with faculty members and students from a technical nursing program, using the World Café technique.

**Results:**

The analyses revealed gaps in the development of digital competencies and in theory-practice integration, associated with the lack of specific preparation for students to use digital tools such as the EHR. Still, participants recognized the importance of this technology in technical nursing training.

**Final considerations:**

Teaching and learning related to the EHR in technical nursing education require continuous, cross-curricular integration of digital competencies into the Political-Pedagogical Project, along with stronger faculty development aligned with the demands of vocational education.

## RESUMEN

**Objetivo:**

Analizar la enseñanza y el aprendizaje de la historia clínica electrónica en la formación técnica en enfermería, identificando debilidades y potencialidades para mejorar el Proyecto Político-Pedagógico (PPP).

**Método:**

Estudio cualitativo, desarrollado mediante análisis documental y estudio de campo con docentes y estudiantes, utilizando la técnica *World* Café.

**Resultados:**

Los análisis mostraron brechas en el desarrollo de competencias digitales y en la integración entre teoría y práctica, asociadas a la ausencia de formación específica del estudiantado para el uso de herramientas digitales como la historia clínica electrónica. Sin embargo, los participantes reconocieron la importancia de la tecnología en el proceso formativo. **Consideraciones finales:**La enseñanza y el aprendizaje de la historia clínica electrónica en la formación técnica en enfermería requieren la integración transversal y continua de las competencias digitales en el PPP y el refuerzo de la formación continuada del profesorado acorde con las demandas de la educación profesional.

## INTRODUCTION

Brazil has approximately 2,965,953 nursing professionals, of whom approximately 27% are registered nurses and 73% are nursing assistants and nursing technicians^(1)^. This large technical-level workforce underscores the importance of examining this group’s training profile.

Technical nursing education is marked by gaps stemming from historical curriculum changes and from the political, economic, and social contexts that shape professional practice. Within this context, the pedagogical project plays a strategic role in curriculum implementation and in legitimizing the body of knowledge on which training is built, as technical education faces the challenge of preparing students for a constantly changing healthcare environment^(2)^. Difficulties in reading, writing, and mathematics, often observed at this educational level, can weaken digital literacy and hinder the effective use of technologies that are essential to clinical practice, such as the electronic health record (EHR), with direct implications for the quality of care.

The National Catalog of Technical Courses (Catálogo Nacional de Cursos Técnicos, CNCT) highlights, in the nursing technician’s professional profile, competencies such as problem solving, effective communication, teamwork, and interdisciplinary practice, as well as proficiency in information and communication technologies (ICTs), conflict management, and professional ethics. The program is structured to cover these competencies over a minimum of 1,200 hours. Developed and maintained by Brazil’s Ministry of Education, the CNCT also underscores the importance of mastering the technologies relevant to each technological field to support students’ progressive professional development^(3)^.

The National Common Core Curriculum (Base Nacional Comum Curricular, BNCC) also emphasizes the need for digital literacy in technical education, guiding the development of competencies that promote the critical and ethical use of technologies^(4)^. Within this framework, upper-secondary technical education seeks to adopt innovative educational programs that prepare students for today’s labor market while adapting to ongoing changes and demands. It also aims to develop skills that enable students to be productive and innovative, taking into account the local context and the educational opportunities available^(5)^.

The use of the electronic health record (EHR) is addressed in Brazil’s Nursing Professional Practice Law and in Federal Nursing Council (Conselho Federal de Enfermagem, COFEN) Resolution No. 754/2024^(6,7)^. These documents highlight the need for computer resources to support care planning according to each professional group’s scope of practice, as well as the preparation required to use them. Beyond their role in point-of-care documentation, EHR entries also generate data that can be used in research, audits, and other processes aimed at improving the quality of health care.

Nursing technicians’ autonomy and knowledge in documenting the care process are therefore essential to ensuring that patients receive comprehensive, evidence-based care^(8)^. In nursing practice, this includes using electronic health systems to document patient information, review medical histories, and enter data on treatment and care provided^(9)^.

Digital literacy encompasses social reading and writing practices that require not only language proficiency but also the learning of technical procedures. Whereas handwriting relies on specific tools and its own directional logic, writing in digital environments requires learning to use devices such as keyboards and mice, as well as becoming familiar with the graphical interfaces through which texts are produced^(10)^. Digital literacy, therefore, refers to the ability to make meaningful use of new digital technologies and to engage in reading and writing practices in digital environments in ways that differ from those performed on paper. A digitally literate individual uses technology to search for, select, modify, evaluate, and understand new information, drawing on a range of digital tools to do so^(11)^.

Students need to develop critical analysis skills and actively participate in technology mediated teaching processes, since interaction in these environments requires knowledge of digital culture, including reading, writing, spoken language, analysis, and linguistic reflection^(12)^.

It is important to emphasize that digital transformation in health care is not limited to the incorporation of new equipment and systems. Above all, it involves building professional competencies aligned with a context of connectivity and networked interaction^(13)^. These competencies are part of nurses’ day-to-day work, but they still need to be adapted to Brazilian hospital settings, especially given technological advances and the need to tailor them to each health care institution’s specific context. Professional training remains fragile in this regard, directly affecting clinical practice^(14)^.

This scenario also extends to undergraduate nursing education, where gaps in training are evident, especially in preparing students to ensure continuity of care in digital environments. When this fluency is not adequately developed during undergraduate education, it needs to be rebuilt through continuing education programs in workplace settings^(15)^.

Teaching students to use electronic health records still poses a major challenge for many nursing programs, largely because opportunities for hands-on training in real clinical settings remain limited. In this context, simulated electronic health records have proven to be an effective strategy because they provide an experiential, learner-centered approach. This methodology allows students to develop and apply informatics knowledge in a safe learning environment before interacting with real patients^(16)^.

During training, the inclusion of technologies such as the EHR can not only strengthen technical skills but also increase students’ familiarity with this tool, helping build competencies such as reading, writing, and critical thinking. In this context, the National Curriculum Guidelines for Upper-Secondary Technical and Vocational Education highlight the need to develop competencies specific to professional practice, but they do not specify how to incorporate digital tools.

Against this backdrop, we ask how faculty members and students in a technical nursing program view the role of the program’s pedagogical project (Projeto Político-Pedagógico, PPP) in developing competencies for EHR use.

## OBJECTIVE

To analyze teaching and learning related to EHR use in a technical nursing program, identifying weaknesses and strengths to inform improvements to the PPP.

## METHOD

### Ethical aspects

The Research Ethics Committee at UNIFESP reviewed and approved the research project. All faculty and students in the technical nursing program who agreed to participate signed a written informed consent form.

### Type of study

This exploratory, descriptive, qualitative, and analytical study was reported in accordance with the Consolidated criteria for reporting qualitative research (COREQ), a checklist that guides reporting and helps improve the quality of qualitative studies^(17)^.

### Methodological procedures

This study combined document analysis with a field study using the World Café technique to explore the conceptions and experiences of faculty and students in a technical nursing program. It was based on the premise that qualitative research is well suited to examining relationships, representations, perceptions, and opinions arising from the ways individuals think, feel, and interact socially^(18)^.

### Study setting

The study was conducted in the technical nursing program of a nonprofit teaching and research institute in a municipality in the eastern part of São Paulo state, Brazil.

### Data sources

Document analysis focused on the PPP, course syllabi, and teaching plans for the courses that make up the curriculum.

In the field stage, participants included faculty and students. Among faculty, all instructors affiliated with the institution’s technical nursing program were considered eligible provided that they had been affiliated with the program for at least six months, taught theoretical courses and/or supervised clinical practicums, and agreed to participate in the study in full. All 16 faculty members met the inclusion criteria and agreed to participate; there were no exclusions or withdrawals in this group.

At the time of data collection, 25 students were regularly enrolled in the second module of the technical nursing program. Students were eligible if they had already completed the mandatory supervised clinical practicum. Those who had not yet begun the practicum or who withdrew from the program during the study period were excluded. All 25 students were eligible. However, after receiving the invitation, only 16 agreed to participate, and there were no withdrawals. All participants were contacted through an invitation letter sent via a messaging app, which included a link to a digital form describing the study objectives and the written informed consent form.

### Data collection and organization

Data were collected in two complementary stages. The first involved document analysis of institutional records and documents, and the second consisted of in-person sessions with faculty and students conducted using the World Café technique.

For the document analysis, the PPP, course syllabi, and teaching plans for the courses comprising the technical nursing program curriculum were reviewed. This stage examined how digital competencies are distributed across the curriculum and whether they are addressed in isolation or integrated into training, especially in courses involving clinical practice. The gathered information was organized into a concept map.

Data from participants were collected using the World Café technique, chosen for its flexibility and its ability to foster collaborative dialogue and collective meaning-making. Structured as small-group discussions organized into successive rounds, the technique promotes the circulation of ideas, the integration of different perspectives, and the construction of shared meanings, making it a valuable resource for research in educational and training contexts^(19,20)^.

Data collection took place in two separate sessions, one with students and the other with faculty, each lasting approximately 1 hour and 30 minutes. The room was arranged to encourage interaction, with tables organized into small groups of four participants and graphic materials available for recording ideas. At the outset, the study objectives and the procedures of the World Café technique were presented. The activity unfolded over four rounds of discussion guided by a semistructured discussion guide with questions tailored to each group. With faculty, the discussion addressed knowledge of the EHR, its integration into classes, and the challenges of teaching it. With students, the discussion explored their understanding of EHR use, their exposure to it throughout the program, perceived difficulties, contributions to professional practice, and suggestions for learning. Each round lasted approximately 10 minutes, during which participants recorded their ideas. At the end of each round, one participant remained at the table as host, while the others rotated among the groups. In the final round, participants returned to their original tables to synthesize and collectively share the recorded ideas.

The material was transcribed, subjected to an initial reading, and analyzed using thematic analysis, resulting in the development of analytical categories. To preserve anonymity, each participant’s name was replaced with an alphanumeric code consisting of the letter F for faculty or S for students, followed by an Arabic numeral indicating the order of participation. The data were recorded and stored on digital memory cards for the researcher’s exclusive use. In accordance with National Health Council Resolution 510/2016^(21)^, the material will be stored for at least five years.

### Data analysis

Data analysis followed a qualitative methodological orientation and was conducted in two complementary stages: document analysis and thematic analysis of the data generated through the World Café technique.

Document analysis was carried out before the field stage to contextualize the technical training setting and identify institutional guidelines, standards, and directions related to EHR use in teaching. Document analysis is important for locating, identifying, organizing, evaluating, and contextualizing information contained in original sources^(22)^. It is also considered a complementary research technique, with advantages such as low cost and minimal interference with participants^(23,24)^.

The selection and analysis of institutional documents, along with the course plan, enabled the identification of key elements of technical nursing training. For this stage, the following guiding documents served as references: the Law of Guidelines and Bases of National Education, the National Common Core Curriculum, Federal Nursing Council standards, the National Catalog of Technical Courses, the National Curriculum Guidelines, and the National Curriculum Parameters.

Field data were generated through the World Café technique with faculty and students in the technical nursing program. The material was transcribed manually and submitted to thematic analysis. The analytic process followed three stages: pre-analysis, involving exhaustive reading of the transcripts and formulation of initial hypotheses; exploration of the material, involving coding and categorization of recording units and meaning units; and treatment of the results, involving the organization of the data into thematic clusters interpreted in light of the study objectives and theoretical framework^(25)^.

Triangulation of documentary data with participants’ accounts enabled a more robust analysis, revealing convergences, weaknesses, and pedagogical possibilities for developing competencies in EHR use in technical nursing education. This methodological integration enriched the findings by broadening understanding of the phenomenon under investigation through different sources and perspectives.

## RESULTS

Based on document analysis of the course syllabi and the PPP, the main competencies related to EHR use across the program’s courses were mapped (Chart 1).

**Chart 1:** Mapping of competencies developed across the curriculum. São José dos Campos, SP, Brazil, 2024

Courses	Identified Competencies
First Aid	Activity recording; communication; nursing documentation.
Public Health	Written communication; nursing documentation; use of computer tools.
Work Process Organization (OPTE)	Activity recording; communication; form completion; use of computer tools.
Supervised Clinical Practicum	No records were identified.
Maternal and Child Health	Communication; activity recording.
Mental Health	Communication; activity recording.
Biological Specimen Collection	Communication; activity recording.
Medication Administration	Activity recording; communication; prescription checking.
Fundamentals of Nursing	Activity recording; technology use in care delivery; documentation of nursing actions.
Urgent and Emergency Care	Activity recording; communication; nursing documentation.
Integrative Project	No records were identified.
Perioperative Nursing	Activity recording; communication; nursing documentation.
Critically Ill Patient Care	Activity recording; communication; prescription checking.

Content related to the use of computer tools appears only in the courses Fundamentals of Nursing, Public Health, and OPTE. Core courses such as Critically Ill Patient Care, Urgent and Emergency Care, and Medication Administration do not include digital technologies in their course content. In addition, key parts of the training process, such as the supervised clinical practicum and the Integrative Project, also contain no documented activities specifically focused on EHR use.

Reading and analysis of the material transcribed and produced during the World Café sessions with students yielded 56 context units and 107 recording units, from which three thematic clusters emerged: Students’ Knowledge of EHR; The Educational Process related to EHR in the program; and Faculty Development from the Students’ Perspective for EHR Teaching. In contrast, analysis of faculty members’ accounts yielded 96 context units and 45 recording units, from which four clusters emerged: Knowledge and Use of EHR in Teaching; Technology and Digital Inclusion; Faculty Development; and Teaching Methodologies and Practices.

Regarding the first thematic cluster, Students’ Knowledge of EHR, most students recognized its benefits, such as greater agility and safer care delivery. However, they emphasized that they were not adequately prepared to use it because it is introduced late in the program and addressed only superficially. Faculty, in turn, recognized EHR as an essential tool for integrating information and promoting patient safety. However, they also pointed to limitations in its teaching, including a lack of faculty training and students’ difficulties with basic computer skills.

*Some students lack a solid foundation in basic computer skills, which makes it harder for us to teach.* (F1)

As for the thematic cluster The Educational Process Related to EHR, students reported that their first hands-on contact with the record occurred directly in the clinical setting, without adequate preparation. They also described difficulties using it, such as limited access to the system, lack of confidence due to unfamiliarity with computers, and a lack of standardization in nursing documentation.

Despite these difficulties, students expressed interest in more practice-oriented training, suggesting the inclusion of specific classes on EHR throughout the program and the use of laboratory simulations to improve learning. They emphasized the importance of activities involving the practical application of the technology, arguing that practice fosters greater autonomy and better prepares them for the labor market. Finally, they expressed the expectation that the program should offer a more integrated learning environment, with simulators and digital systems in place from the outset.

In the thematic cluster Faculty Development for Teaching EHR, students reported that hands on experience with faculty in high-acuity units, such as the ICU and the operating room, was enriching. In these settings, faculty actively encouraged direct patient contact and the use of digital resources, which helped consolidate students’ skills.

*The instructors who stayed with us taught us a lot, especially in the ICU. Every day they told us to have that contact. However, when I did my practicum on the night shift, access wasn’t the same.* (F1)

Some students reported that guidance on EHR use during their practicums was complemented-or even replaced-by support from health care institution staff, such as nursing technicians and other employees.

*In my case, I learned more about how to use EHR from the technicians who walked me through how to document.* (F2)

*The staff at the institution answered our questions.* (F4)

Within this same thematic cluster, faculty expressed differing views on how prepared they felt to teach EHR. For some instructors, hands-on experience and prior exposure to the system in clinical settings were key to their sense of preparedness. Others, however, who had not had that experience, reported difficulty teaching EHR, reinforcing the need for additional, specific training.

*We cover it in class because, at some point, we had already come across EHR ourselves, but we still need updates.* (F15)

Some faculty also pointed out that students do not always learn to use the system from their instructors, but rather from other health care professionals during their practicums.

[We need] *technical training so instructors can better understand the system, because students always have a lot of questions, and when the instructor cannot help, it creates a bad impression.* (F8)

*We do not feel prepared to teach it: we lack training and guidance. We do not have difficulty with computers.* (F7)

Regarding Technology and Digital Inclusion, faculty emphasized that students’ limited grasp of basic information and communication terminology poses a major challenge to adapting to health technologies, especially as systems become increasingly computerized.

*Some students lack a solid foundation in computer skills, which makes it harder for us to teach.* (F5)

*We struggle to develop the reasoning skills of students who have difficulty with digital inclusion.* (F4)

These accounts indicate that many students struggle with essential computer skills, which directly affects how these resources are taught in the training of future professionals. They also point to gaps in digital literacy, such as difficulty locating letters on a keyboard or lack of familiarity with computers, suggesting that some students have not had adequate exposure to basic technologies.

*We have students who do everything by hand, so they have difficulty with computers. The generation that uses smartphones all the time-the classic “scroll up, scroll down”-does not know how to type, use, or even turn on a computer.* (F10)

For faculty, the contrast between students who use smartphones and those who need to work with computers reveals a methodological challenge. Frequent use of mobile devices does not ensure proficiency in tasks involving computers and more complex systems such as EHR, creating a need for pedagogical intervention to develop these skills.

Faculty also highlighted the diversity of systems across institutions, which makes learning and continued use of the tool more difficult. This lack of standardization undermines students’ autonomy and hands-on learning, limiting their ability to explore and become familiar with EHR in real-world work settings.

Faculty further emphasized the need for EHR teaching to run throughout every stage of students’ training, aligning the curriculum with continuous, contextualized use of the system, from the first modules through the end of the program.

*I believe we need to build this awareness from the beginning to the end of the program, weaving it throughout the curriculum so our students can reach a more even level of knowledge during training.* (F15)

Finally, some faculty reported that their preparedness to teach EHR is tied not only to technical training but also to the experience accumulated throughout their professional trajectories, showing that pedagogical competence develops continuously and depends on both personal experience and technical updating.

*When people ask whether we are prepared to teach, we bring to it the preparation we have gained through life, according to each professional’s knowledge over the course of their trajectory.* (F10)

Regarding Teaching Methodologies and Practices, faculty highlighted several challenges in technical education, especially gaps in Portuguese and mathematics, as well as in digital literacy. Even with specific courses in the program, students who have been away from school for long periods face difficulties, which calls for a more tailored approach to teaching.

To address these difficulties, faculty proposed using active learning strategies to encourage students’ autonomy and responsibility in the training process. They also emphasized the need to increase the course hours devoted to computing and EHR, implement electronic system simulators in class, and offer workshops and extra classes to strengthen students’ ability to work with EHR.

## DISCUSSION

Document analysis of the PPP and teaching plans revealed gaps in the development of competencies in digital literacy and EHR use throughout technical nursing education. This finding is consistent with studies showing that this absence may compromise the development of the competencies that future nursing technicians need to work in computerized health care settings^(13)^.

Despite some isolated initiatives to build digital competencies, important barriers to consolidating them in the curriculum remain. These limitations stem from the absence of integrated pedagogical planning linking theory and practice, resulting in fragmented teaching. In this sense, the lack of pedagogical planning makes it harder for students to understand how informatics applies to their professional practice^(26)^.

Document analysis also revealed a lack of clear guidance in the PPP and course syllabi on EHR use, highlighting the weak integration of ICTs into training. This finding underscores structural gaps that compromise the effectiveness of educational processes and call for revisions to pedagogical policies(24). Some courses address digital resources, but only in isolated ways rather than through a cross-curricular approach, with no coordination across the program’s different modules. Similarly, a study in higher education points to the same lack of coordination around digital tools, given the inadequate preparation of newly graduated students^(16)^.

Both faculty and students found the quality of EHR teaching inconsistent, leading to frustration and learning difficulties. This observation suggests that not all instructors have the same level of command of the tool. Another relevant issue is the difficulty of teaching students the logic of the integrated care network and access to information. This fragmented understanding points to an obstacle in the training process and to the need for stronger pedagogical planning to prepare students to work in multidisciplinary contexts that require knowledge integration and collaboration across disciplines^(27)^.

The accounts highlight the need for ongoing faculty training so that instructors can keep up with updates to digital systems and integrate them into training. Although many faculty members do not have difficulty with computers, the lack of specific training in EHR use may pose a significant barrier to pedagogical practice, as evidenced by both student and faculty accounts. Faculty development, therefore, needs to address digital literacy, going beyond the mere use of tools to promote critical, collaborative, and contextualized pedagogical practice^(13)^.

Both students and faculty identified curricular fragmentation and the late introduction of EHRs as factors that hindered learning and the use of the system. This scenario shows that progress in digital transformation in health care requires the development of professional competencies aligned with connectivity and networked work, which, in turn, requires continuous training adapted to institutions’ technological realities^(13)^.

The results show that digital literacy remains a challenge for some students, especially those who are less familiar with computers. This finding is consistent with a study that understands digital literacy as extending beyond technical mastery to include the ability to interpret, understand, and produce information^(10)^. In the present study, the difficulties observed in EHR use indicate that digital technology-related skills are not always developed linearly throughout the training process, reinforcing the need for innovative pedagogical strategies that promote meaningful, critical engagement with these technologies in technical nursing education^(28)^.

In the teaching context, the need for specific training in the use of digital tools was also evident. Although many faculty members have clinical experience, gaps remain in pedagogical preparation for working with digital health systems. This reality is consistent with findings from a study showing insufficient preparation of both faculty and students for digital health and a mismatch between training and professional practice^(26)^. Faculty in technical nursing programs face challenges related to diverse student profiles, lack of interest, and students’ weak initial preparation; these difficulties call for critical reflection on educational practices and ongoing faculty development^(29)^. In addition, the lack of standardization in the systems used across institutions increases the complexity of teaching, requiring students to constantly adapt and making it harder to consolidate practical knowledge^(26)^.

From another angle, there is broad recognition of EHR as an essential tool for patient safety and for information integration in care delivery. COFEN Resolution No. 754/2024 addresses the use of EHR and digital platforms in nursing practice in Brazil, establishing guidelines for secure, confidential, and ethical recordkeeping of care information^(7)^. It underscores nursing professionals’ responsibility when using electronic records as tools for management and for improving the quality of care, highlighting the need to develop these competencies from initial training onward^(1)^.

Accordingly, technological difficulties in EHR use affect the work process of the technical nursing team, indicating that digital competencies aligned with labor market demands need to be integrated across technical training, thereby strengthening digital literacy, critical thinking, and professional autonomy^(30)^. The results, therefore, point to the importance of revising the PPP by incorporating interdisciplinary pedagogical practices that promote the development of students’ digital competencies, high-fidelity simulation activities as a teaching strategy, and ongoing faculty development so that training keeps pace with the demands of today’s digital health landscape^(31)^.

### Study limitations

This study has methodological limitations. It was conducted at a single institution; in addition, it used a qualitative approach, which limits the generalizability of the findings to other populations and contexts.

### Contributions to practice

The findings highlight gaps in nursing technicians’ training in the use of digital systems such as EHR. They also underscore the importance of ongoing faculty development to strengthen pedagogical practice and to integrate digital technologies effectively into teaching.

## FINAL CONSIDERATIONS

The findings suggest that technical nursing education has made isolated advances, but the systematic inclusion of digital competencies related to tools such as EHR remains insufficiently established. The development of these competencies is still fragmented, revealing shortcomings in pedagogical planning and in the effective integration of digital technologies into the curriculum. Among the weaknesses identified are the late introduction of EHR in the training pathway, the limited use of simulated experiences before students encounter the system in clinical practicums, and insufficient theory-practice integration. The diversity of electronic systems used across health care institutions, together with the lack of specific complementary training, also makes it harder for students and faculty to gain confidence and proficiency in using EHR. At the same time, one important strength was faculty recognition of EHR as a relevant pedagogical resource. This perception provides a favorable starting point for strengthening educational practices related to digital technologies, provided those practices are supported by consistent training processes.

Accordingly, the recommendations arising from these findings include revising the PPP so that EHR teaching, aligned with digital competencies, is addressed in a cross-curricular and continuous way throughout the program. Expanding course hours devoted to computing and health technologies, implementing electronic simulators, and offering workshops and supplementary activities may also help consolidate digital literacy.

Ongoing faculty development is also essential, since pedagogical competence develops continuously and depends on both technical updating and critical reflection on practice. Strengthening digital literacy in technical nursing education requires coordination among institutional policies, pedagogical practices, and investment in faculty development so that professional training keeps pace with technological change and responds to the contemporary demands of health care.

## Data Availability

Research data is only available upon request.
