# Supplementary material for: Teaching the electronic health record in a technical nursing program
Source: Rev Bras Enferm. 2026 Oct 16;79:e20250641. doi: 10.1590/0034-7167-2025-0641 (PMC13679168; doi:10.1590/0034-7167-2025-0641)
Supplement: Translation

# Translation: O ensino do prontuário eletrônico do paciente no curso técnico em Enfermagem

**DOI:** 10.1590/0034-7167-2025-0641pt

**Authors:** Renata da Paixão Pereira, Christine Barbosa Betty, Nildo Alves Batista

**Affiliations:** I Instituto de Ensino e Pesquisa da Santa Casa de São José dos Campos. São José dos Campos, SP, Brasil; II Universidade Federal de São Paulo. São Paulo, SP, Brasil

**Keywords:** Educação Técnica em Enfermagem, Letramento em Saúde, Prontuário Eletrônico do Paciente, Tecnologia em Saúde, Ensino.

## Abstract

**Objetivo:**

Analisar o ensino e a aprendizagem sobre o prontuário eletrônico do paciente no curso técnico em Enfermagem, identificando fragilidades e potencialidades para subsidiar melhorias no Projeto Político-Pedagógico.

**Método:**

Pesquisa qualitativa, exploratória e descritiva, desenvolvida mediante estudo documental e estudo de campo com docentes e estudantes do curso técnico em Enfermagem, utilizando a técnica *World* Café.

**Resultados:**

As análises indicaram lacunas no desenvolvimento de competências digitais e na integração entre teoria e prática, associadas à ausência de formação discente específica para o uso de ferramentas digitais, como o prontuário eletrônico do paciente. Contudo, os participantes reconheceram a importância dessa tecnologia associado ao processo formativo.

**Considerações finais:**

O ensino e aprendizagem sobre o prontuário eletrônico do paciente na formação técnica em Enfermagem requer a integração transversal e contínua das competências digitais no Projeto Político-Pedagógico, aliada ao fortalecimento da formação docente em consonância com as demandas da educação profissional.

## INTRODUÇÃO

A força de trabalho da enfermagem no Brasil é de aproximadamente 2.965.953 profissionais, dos quais se estima que 27% são enfermeiros e 73% são auxiliares e técnicos em enfermagem^(1)^. Esse expressivo número de profissionais de nível técnico destaca a importância de analisar o perfil de formação dessa categoria.

O ensino técnico em Enfermagem é marcado por lacunas relacionadas às mudanças curriculares históricas e aos contextos políticos, econômicos e sociais que influenciam o exercício profissional. Nesse cenário, o projeto pedagógico assume função estratégica na implantação do currículo e na legitimação dos saberes formativos, em uma formação técnica que enfrenta o desafio de preparar estudantes para um contexto de saúde em constante transformação^(2)^. As dificuldades em leitura, escrita e matemática, frequentemente observadas nesse nível de ensino, repercutem em fragilidades no letramento digital e impactam o uso qualificado de tecnologias essenciais à prática assistencial, como o prontuário eletrônico do paciente, com implicações diretas na qualidade do cuidado.

O Catálogo Nacional de Cursos Técnicos (CNCT) ressalta, no perfil profissional do técnico em enfermagem, competências como a resolução de situações-problema, comunicação eficaz, trabalho em equipe e interdisciplinaridade, além do domínio de tecnologias da informação e comunicação, gestão de conflitos e ética profissional. O curso é estruturado para contemplar essas competências em uma carga horária mínima de 1.200 horas. Instituído e organizado pelo Ministério da Educação, o CNCT reforça ainda a importância do domínio intelectual das tecnologias pertinentes a cada eixo tecnológico, visando ao progressivo desenvolvimento profissional do estudante^(3)^.

A Base Nacional Comum Curricular (BNCC) também enfatiza a necessidade de letramento digital na educação técnica, orientando o desenvolvimento de competências que incentivem o uso crítico e ético das tecnologias^(4)^. Nesse sentido, a formação técnica de nível médio busca implementar programas educacionais inovadores que preparem os estudantes para o mercado de trabalho atual, adaptando-se às mudanças e demandas constantes; também, visa desenvolver habilidades que capacitem os estudantes a serem produtivos e inovadores, considerando o contexto local e as oportunidades de ensino disponíveis ^(5)^.

O uso do Prontuário Eletrônico do Paciente (PEP) é tratado pela Lei do Exercício Profissional da Enfermagem e pela Resolução do Conselho Federal de Enfermagem (COFEN) Nº 754/2024^(6,7)^. Esses documentos destacam a exigência de recursos computacionais para o planejamento das ações, de acordo com as competências de cada grupo profissional, bem como o preparo necessário para o uso desse recurso. Relevantes no cuidado imediato, os registros no PEP contribuem para a geração de dados que podem ser usados em pesquisas, auditorias e outros processos voltados à melhoria da qualidade da assistência à saúde.

A autonomia e o conhecimento do técnico em enfermagem no registro do processo de cuidado são, portanto, essenciais para garantir que o paciente receba uma atenção integral e baseada em evidências ^(8)^. Na prática de enfermagem, isso envolve o uso de sistemas eletrônicos de saúde para registrar informações do paciente, acessar históricos médicos e inserir dados de tratamento e do cuidado prestado ^(9)^.

O letramento digital compreende práticas sociais de leitura e escrita que envolvem, além do domínio da linguagem, o aprendizado de procedimentos técnicos. Enquanto a escrita manual requer instrumentos específicos e direcionalidade própria, a escrita em ambientes digitais demanda a aprendizagem do uso de dispositivos como teclado e mouse, bem como a familiarização com as interfaces gráficas que mediam a produção textual ^(10)^. O termo letramento digital, portanto, refere-se à capacidade de se apropriar das novas tecnologias digitais e de realizar práticas de leitura e escrita em ambientes digitais, de forma distinta daquelas realizadas no papel. O letrado digital interage com as tecnologias para pesquisar, selecionar, modificar, avaliar e compreender novas informações, utilizando diferentes ferramentas digitais para isso ^(11)^.

É essencial que o estudante desenvolva habilidades de análise crítica e participação ativa em processos de ensino mediados pelas tecnologias digitais, uma vez que a interação nesses ambientes exige conhecimentos relacionados à cultura digital, envolvendo aspectos como leitura, escrita, oralidade, análise e reflexão linguística ^(12)^.

Importa frisar que o cenário de transformação digital na saúde não se limita à incorporação de novos equipamentos e sistemas, mas envolve, sobretudo, a construção de competências profissionais alinhadas a um contexto de conectividade e interação em rede ^(13)^. Essas competências são aplicadas no cotidiano profissional em enfermagem, porém ainda demandam adaptações para o contexto hospitalar brasileiro, especialmente diante do avanço tecnológico e da necessidade de adequações à realidade específica de cada instituição de saúde. Observa-se uma fragilidade na formação dos profissionais, o que repercute diretamente no exercício prático da profissão ^(14)^.

Esse cenário estende-se para a formação dos graduandos em Enfermagem, em que se observam lacunas na qualificação, principalmente no tocante à preparação para a continuidade do cuidado em ambientes digitais. Quando insuficiente durante a graduação, essa fluência precisa ser resgatada por meio de programas de educação continuada nos ambientes de trabalho ^(15)^.

Ensinar estudantes sobre o uso de prontuários eletrônicos de saúde ainda representa um desafio significativo para muitos cursos de Enfermagem, principalmente devido à escassez de oportunidades de treinamento prático em ambientes clínicos reais. Nesse sentido, a utilização de prontuários eletrônicos simulados tem se mostrado uma estratégia eficaz, ao proporcionar uma abordagem experiencial e centrada no aluno. Essa metodologia permite que os estudantes desenvolvam e apliquem conhecimentos de informática em um ambiente de aprendizagem seguro, antes do contato com pacientes reais ^(16)^.

Durante a formação, a inclusão de tecnologias como o PEP poderá não só aprimorar a habilidade técnica, mas também aproximar o estudante desse recurso, possibilitando o fortalecimento de competências como leitura, escrita e pensamento crítico. Nesse contexto, as Diretrizes Curriculares Nacionais para a Educação Profissional Técnica de Nível Médio evidenciam a necessidade de desenvolver competências específicas para a prática profissional, mas não detalham a inclusão de ferramentas digitais.

Diante de tal cenário, questionamos como professores e estudantes de um curso técnico em Enfermagem enxergam o papel do Projeto Político-Pedagógico (PPP) no desenvolvimento de competências para o uso do PEP.

## OBJETIVO

Analisar o ensino e aprendizagem sobre o prontuário eletrônico do paciente no processo formativo do curso técnico em Enfermagem, identificando fragilidades e potencialidades para subsidiar melhorias no Projeto Político-Pedagógico.

## MÉTODO

### Aspectos éticos

O projeto da pesquisa foi avaliado e aprovado pelo Comitê de Ética em Pesquisa (CEP) da UNIFESP. Todos os professores e estudantes do curso técnico em Enfermagem que aceitaram participar da pesquisa assinaram o Termo de Consentimento Livre e Esclarecido (TCLE).

### Tipo de estudo

Trata-se de um estudo exploratório, descritivo, qualitativo e analítico, conduzido em conformidade com o *Consolidated criteria for reporting qualitative research* (COREQ), checklist que orienta o relato e contribui para a qualidade dos estudos qualitativos^(17)^.

### Procedimentos metodológicos

Este trabalho foi desenvolvido com base em uma pesquisa documental e em um estudo de campo por meio da técnica *World* Café, explorando as concepções e experiências dos sujeitos envolvidos (docentes e estudantes) de um curso técnico em Enfermagem. Partiu-se do pressuposto de que a abordagem qualitativa se aplica ao estudo das relações, representações, percepções e opiniões resultantes da forma como o indivíduo pensa, sente e interage socialmente ^(18)^.

### Cenário do estudo

A pesquisa foi realizada no curso técnico em Enfermagem de um instituto de ensino e pesquisa sem fins lucrativos de um município localizado na região leste do estado de São Paulo.

### Fonte de dados

A análise documental teve como foco o PPP, ementas e planos de ensino das disciplinas que compõem a matriz curricular.

Na etapa de campo, participaram do estudo docentes e estudantes. Dentre os docentes, foram considerados elegíveis todos os professores vinculados ao curso técnico em Enfermagem da instituição, desde que possuíssem vínculo mínimo de seis meses, atuassem em disciplinas teóricas e/ou na supervisão de estágios e aceitassem participar integralmente do estudo. Todos os 16 docentes atenderam às condições de inclusão e aceitaram participar, não havendo exclusões ou desistências nesse grupo.

Quanto aos estudantes, havia 25 alunos regularmente matriculados no 2º módulo do curso técnico em Enfermagem no momento da coleta de dados. Como critérios de inclusão, foram considerados estudantes que já tivessem vivenciado o estágio supervisionado obrigatório. Foram excluídos aqueles que ainda não haviam iniciado o estágio ou que cancelaram a matrícula durante o período da pesquisa. Todos os 25 estudantes mostraram-se elegíveis. Contudo, após o convite para participação, apenas 16 aceitaram integrar o estudo, não havendo registros de desistência. Todos os participantes foram contatados por meio de carta-convite enviada via aplicativo, contendo link para um formulário digital com a apresentação dos objetivos da pesquisa e o Termo de Consentimento Livre e Esclarecido (TCLE).

### Coleta e organização dos dados

A coleta de dados foi realizada em duas etapas complementares: a primeira consistiu no estudo documental, voltado à busca de informações em registros e documentos institucionais; e a segunda envolveu encontros presenciais com professores e estudantes, conduzidos por meio da técnica *World* Café.

Para o estudo documental, foram consultados o PPP, ementas e planos de ensino das disciplinas que compõem a matriz curricular do curso técnico em Enfermagem. Essa etapa visou verificar como as competências digitais estão distribuídas e se são tratadas de maneira isolada ou integradas à formação, especialmente nas disciplinas que envolvem práticas assistenciais. As informações obtidas foram sistematizadas por meio de um mapa conceitual.

A coleta de dados com os sujeitos da pesquisa foi realizada por meio da técnica *World* Café, escolhida por seu caráter flexível e por favorecer o diálogo colaborativo e a construção coletiva de sentidos. Estruturada em discussões com pequenos grupos organizados em rodadas sucessivas, a técnica promove a circulação de ideias, a integração de diferentes perspectivas e a produção de sentidos compartilhados, configurando-se como um recurso relevante para investigações em contextos educacionais e formativos ^(19,20)^.

A coleta de dados ocorreu em dois encontros distintos: um com estudantes e outro com docentes, ambos com duração aproximada de uma hora e trinta minutos. O ambiente foi organizado para favorecer a interação, com mesas dispostas em pequenos grupos de quatro participantes e materiais gráficos para registro das ideias. Inicialmente, foram apresentados os objetivos da pesquisa e os procedimentos da técnica *World* Café. A dinâmica se deu em quatro rodadas de discussão, orientadas por roteiro semiestruturado, com questões específicas para cada grupo. Com os docentes, abordaram-se conhecimentos sobre o PEP, sua integração às aulas e os desafios do ensino. Com os estudantes, investigaram-se a compreensão do uso do PEP, o contato ao longo do curso, dificuldades percebidas, contribuições para a prática profissional e sugestões para o aprendizado. Cada rodada teve duração aproximada de dez minutos, com registro das ideias pelos participantes. Ao final de cada rodada, um participante permaneceu como anfitrião da mesa, enquanto os demais realizaram rodízio entre os grupos. Na rodada final, os participantes retornaram às mesas iniciais para síntese e compartilhamento coletivo dos registros.

Os dados produzidos foram transcritos, submetidos à leitura flutuante e analisados por meio da análise temática, resultando na construção de categorias analíticas. Para preservar o anonimato, o nome de cada participante foi substituído por um código alfanumérico, composto pela letra P (professores) ou pela letra E (estudantes) seguida de um numeral cardinal indicando a ordem da entrevista. Os dados foram gravados e armazenados em cartões digitais, de uso exclusivo da pesquisadora. Conforme a Resolução 510/2016 do CNS ^(21)^, o tempo de armazenamento do material será de pelo menos cinco anos.

### Análise dos dados

A análise dos dados seguiu a orientação metodológica qualitativa, tendo sido conduzida em duas etapas complementares: análise documental e análise temática de dados gerados com base na técnica *World* Café.

A análise documental foi realizada antes do estudo de campo, visando contextualizar o cenário da formação técnica e identificar diretrizes, normas e orientações institucionais relacionadas ao uso do PEP no ensino. Destaca-se a importância da análise documental na localização, identificação, organização, avaliação e contextualização de informações presentes nas fontes originais^(22)^. A análise documental também é considerada como técnica complementar de investigação, com vantagens como o baixo custo e a não interferência nos sujeitos da pesquisa^(23,24)^.

A seleção e análise dos documentos institucionais e do plano de curso possibilitaram a identificação de elementos-chave da formação técnica em Enfermagem. Para essa etapa, foram usados como referência os seguintes documentos orientadores: Lei de Diretrizes e Bases da Educação Nacional; Base Nacional Curricular Comum; Normas do Conselho Federal de Enfermagem; Catálogo Nacional dos Cursos Técnicos; Diretrizes Curriculares Nacionais e Parâmetros Curriculares Nacionais.

Os dados de campo foram obtidos por meio da técnica *World* Café com docentes e estudantes do curso técnico em Enfermagem. Foram transcritos manualmente e submetidos à análise temática. O processo analítico seguiu três etapas: pré-análise, com leitura exaustiva das transcrições e formulação de hipóteses iniciais; exploração do material, com codificação e categorização das unidades de registro e núcleos de sentido; e tratamento dos resultados, com a organização dos dados em núcleos temáticos interpretados à luz dos objetivos e do referencial teórico da pesquisa ^(25)^.

A triangulação entre os dados documentais e os relatos dos participantes possibilitou uma análise mais sólida, revelando convergências, fragilidades e possibilidades pedagógicas relacionadas ao desenvolvimento de competências no uso do PEP para o ensino técnico em enfermagem. Essa integração metodológica permitiu enriquecer os achados, ampliando a compreensão do fenômeno investigado por meio de diferentes fontes e perspectivas.

## RESULTADOS

Com base na análise documental das ementas e do PPP, foi realizado um levantamento das principais competências para o manuseio do PEP presentes nas disciplinas do curso (Quadro 1).

**Quadro 1:** Mapeamento das competências desenvolvidas nos componentes curriculares. São José dos Campos, SP, Brasil, 2024

Componentes curriculares	Competências identificadas
Primeiros Socorros	Registro de atividades; comunicação; anotação de enfermagem.
Saúde Coletiva	Comunicação escrita; anotação de enfermagem; uso de ferramentas de informática.
Organização do Processo de Trabalho (OPTE)	Registro de atividades; comunicação; preenchimento de formulários; uso de ferramentas de informática.
Estágio	Não foram encontrados registros.
Saúde Materno-Infantil	Comunicação; registro de atividades.
Saúde Mental	Comunicação; registro de atividades.
Coleta de Materiais Biológicos	Comunicação; registro de atividades.
Administração de Medicamentos	Registro de atividades; comunicação; checagem da prescrição.
Fundamentos de Enfermagem	Registro de atividades; tecnologia aplicada ao cuidado; registro de ações de enfermagem.
Urgência e Emergência	Registro de atividades; comunicação; anotação de enfermagem.
Projeto Integrador	Não foram encontrados registros.
Enfermagem Perioperatória	Registro de atividades; comunicação; anotação de enfermagem.
Paciente Crítico	Registro de atividades; comunicação; checagem da prescrição

Observa-se que os conteúdos sobre o uso de ferramentas de informática estão presentes somente nas disciplinas Fundamentos de Enfermagem, Saúde Coletiva e OPTE. Disciplinas centrais, como Paciente Crítico, Urgência e Emergência e Administração de Medicamentos, não incluem o uso de tecnologias digitais em seus conteúdos. Além disso, momentos importantes da formação, como o estágio supervisionado e o Projeto Integrador, também não apresentam registros de atividades específicas voltadas ao uso do PEP.

A leitura e análise do material transcrito e produzido no *World* Café com os estudantes possibilitaram a identificação de 56 unidades de contexto com 107 unidades de registro, das quais emergiram 3 núcleos temáticos: O conhecimento dos estudantes sobre o PEP; Processo educativo a respeito do PEP no curso; e A formação docente na perspectiva dos estudantes para o ensino do PEP. Por outro lado, a análise das falas dos professores permitiu identificar 96 unidades de contexto com 45 unidades de registro, das quais emergiram 4 núcleos: Conhecimento e uso do PEP no ensino; Tecnologia e inclusão digital; Formação docente; e Metodologias e práticas de ensino.

Quanto ao primeiro núcleo temático, Conhecimento dos estudantes sobre o PEP, a maioria deles reconheceu os benefícios, como agilidade e segurança na assistência, mas destacou a falta de preparo para utilizá-lo adequadamente devido à sua abordagem tardia e superficial no curso. Os professores, por sua vez, reconheceram o PEP como uma ferramenta essencial para integrar as informações e promover a segurança do paciente. No entanto, também apontaram limitações em relação ao seu ensino, como a falta de capacitação docente e as dificuldades dos estudantes com habilidades básicas de informática.

*Existem estudantes que não têm um conhecimento sólido de informática, o que nos dificulta na hora de ensinar.* (P1)

Quanto ao núcleo temático “Processo educativo a respeito do PEP”, os estudantes relataram que o primeiro contato prático com o prontuário ocorreu diretamente no campo clínico, sem preparo adequado. Também reportaram desafios no manuseio desse recurso, como dificuldade de acesso ao sistema, insegurança no uso por falta de familiaridade com computadores e falta de padronização nas anotações.

Apesar das dificuldades, os estudantes demonstraram interesse em uma formação mais prática, sugerindo a inclusão de aulas específicas sobre o PEP ao longo do curso e a realização de simulações em laboratório para melhorar a aprendizagem. Eles defenderam a importância de atividades que envolvam a aplicação da tecnologia, argumentando que a prática promove maior autonomia e preparo para o mercado de trabalho. Por fim, a expectativa dos estudantes foi de que o curso ofereça um ambiente mais integrado, com o uso de simuladores e sistemas digitais desde o início.

No núcleo temático “A formação docente para o ensino do PEP”, os estudantes revelaram que a experiência prática com professores em setores de alta complexidade, como a Unidade de Terapia Intensiva (UTI) e o Centro Cirúrgico, foi enriquecedora. Nessas áreas, os docentes incentivaram ativamente o contato direto com os pacientes e a utilização de recursos digitais, contribuindo para a consolidação das habilidades dos estudantes.

*Os professores que ficaram com a gente até ensinaram bastante, principalmente na UTI. Todo dia falava para ter esse contato. Mas quando fazia estágio à noite, o acesso não era o mesmo.* (E1)

Alguns estudantes relataram que a orientação sobre o uso do PEP nos estágios foi complementada, ou até substituída, pelo apoio de profissionais da instituição de saúde, como técnicos de enfermagem e funcionários do local.

*No meu caso, aprendi a mexer mais com o PEP com os técnicos que me pegaram pela mão para ensinar a anotar.* (E2)

*Dúvidas eram sanadas pelos profissionais da instituição.* (E4)

Ainda nesse mesmo núcleo temático, os professores expressaram percepções divergentes sobre a preparação para o ensino do PEP. Observa-se que a experiência prática e o contato prévio com o sistema em ambientes assistenciais foram determinantes para o sentimento de preparo de alguns docentes. Já outros, que não tiveram essa vivência, relataram dificuldades para ensinar sobre o PEP, reforçando a necessidade de formação específica complementar.

*Abordamos em aula, pois em algum momento já tivemos nos defrontado com o PEP, porém precisamos de atualizações*. (P15)

Alguns docentes apontam também que nem todos os estudantes aprendem o uso do sistema com seus professores, mas, sim, com outros profissionais de saúde durante os estágios.

[É preciso] *o treinamento técnico para os professores entenderem melhor o sistema, pois, perante os estudantes, sempre existe muita dúvida; e quando o professor não consegue auxiliar,* [isso] *causa uma má impressão.* (P8)

*Não nos sentimos preparados para ensinar: falta treinamento e orientação. Não temos dificuldade com informática.* (P7)

Em relação a “Tecnologia e inclusão digital”, os professores destacaram que a falta de conhecimento básico dos estudantes sobre a terminologia de informação e comunicação representa um desafio significativo na adaptação às tecnologias de saúde, especialmente com a crescente informatização dos sistemas.

*Existem estudantes que não têm um conhecimento sólido de informática, o que nos dificulta na hora de ensinar.* (P5)

*Não conseguimos desenvolver o raciocínio desse estudante com dificuldade na inclusão digital.* (P4)

As falas apontam que muitos estudantes apresentam dificuldades com habilidades essenciais de informática, o que impacta diretamente o ensino desses recursos na formação dos futuros profissionais. Além disso, indicam lacunas no letramento digital, como a dificuldade de localizar letras no teclado ou a falta de familiaridade com computadores, sugerindo que alguns estudantes não tiveram uma exposição adequada a tecnologias básicas.

*Temos estudantes que fazem tudo manualmente, então tem essa dificuldade com o computador. A geração que usa muito o smartphone* [o famoso “rola para cima, rola para baixo”] *não sabem digitar, manusear ou ligar o computador.* (P10)

Para os docentes, o contraste entre estudantes que utilizam smartphones e aqueles que precisam lidar com computadores revela um desafio metodológico. O uso frequente de dispositivos móveis não garante a proficiência em tarefas que envolvem o uso de computadores e sistemas mais complexos, como o PEP, gerando uma necessidade de intervenção pedagógica para desenvolver essas habilidades.

Os professores destacaram também a diversidade de sistemas em diferentes instituições, o que dificulta o aprendizado e a continuidade no uso da ferramenta. Essa falta de padronização prejudica a autonomia e o aprendizado prático dos estudantes, restringindo sua capacidade de explorar e se familiarizar com o PEP no contexto real de trabalho.

Os professores ressaltaram ainda a necessidade de que o ensino do PEP permeie todas as etapas da formação do estudante, alinhando o currículo ao uso contínuo e contextualizado do sistema, desde os primeiros módulos até a finalização do curso.

*Acredito que precisamos ter uma conscientização do início ao fim do curso, fazendo um entrelaçamento com o objetivo de equiparar o conhecimento do nosso estudante na formação.* (P15)

Por fim, alguns professores destacaram que seu preparo para o ensino do PEP está ligado não apenas à formação técnica, mas também à experiência acumulada ao longo de suas trajetórias profissionais, evidenciando que o desenvolvimento da competência pedagógica é contínuo e depende tanto da vivência pessoal quanto da atualização técnica.

*Quando nos perguntam se estamos preparados para ensinar, trazemos um preparo da vida, de acordo com o conhecimento de cada profissional ao longo de sua trajetória.* (P10)

Quanto às “Metodologias e práticas de ensino”, os professores destacaram diversos desafios enfrentados no ensino técnico, especialmente relacionados à defasagem nas áreas de português e matemática, além do letramento digital. Mesmo com disciplinas específicas no curso, estudantes com longos períodos fora da escola enfrentam dificuldades, o que demanda uma abordagem mais personalizada para o ensino.

Para enfrentar essas dificuldades, os professores propõem o uso de metodologias ativas de ensino, incentivando a autonomia e a responsabilidade dos estudantes no processo de formação. Além disso, destacam a necessidade de aumentar a carga horária destinada a disciplinas voltadas à informática e ao PEP, implementar simuladores de sistemas eletrônicos nas aulas e realizar oficinas e aulas extras para reforçar o manejo do PEP.

## DISCUSSÃO

A análise documental do PPP e dos planos de ensino revelou lacunas no desenvolvimento de competências relacionadas ao letramento digital e ao manejo do PEP ao longo da formação técnica em enfermagem. Além disso, problematiza um estudo no qual essa ausência pode comprometer a constituição das competências necessárias para a atuação dos futuros técnicos em enfermagem em ambientes de saúde informatizados ^(13)^.

Apesar de algumas iniciativas pontuais voltadas à aquisição de competências digitais, ainda persistem barreiras relevantes à sua consolidação nos currículos. Tais limitações decorrem da ausência de um planejamento pedagógico integrado que articule teoria e prática, resultando em uma fragmentação do ensino. Nesse sentido, a carência de planejamento pedagógico traduz-se em dificuldade para o estudante compreender a prática da informática em sua atuação ^(26)^.

A análise documental revelou a carência de diretrizes claras no PPP e nas ementas para o uso do PEP, refletindo a fragilidade da inserção das TICs de maneira integrada na formação. Esse achado reforça as lacunas estruturais que comprometem a efetividade dos processos educacionais e demandam revisão das políticas pedagógicas ^(24)^. Há disciplinas que abordam o uso de recursos digitais, porém o fazem de forma pontual e não transversal, sem articulação entre os diferentes módulos do curso. Da mesma forma, um estudo realizado no ensino superior aponta para essa desarticulação em relação a ferramentas digitais considerando a falta de preparo dos estudantes recém-formados ^(16)^.

Tanto docentes quanto estudantes julgaram inconsistente a qualidade do ensino sobre o PEP, o que gera frustração e dificuldades no aprendizado. Essa percepção sugere que nem todos os professores têm o mesmo nível de domínio sobre a ferramenta. Outro ponto relevante é a dificuldade de ensinar aos estudantes a lógica da rede integrada de cuidado e o acesso às informações. Esse entendimento fragmentado revela um obstáculo no processo formativo e a necessidade de aprimorar o planejamento pedagógico, visando preparar os estudantes para atuar em contextos multidisciplinares, com integração de conhecimentos e colaboração entre diferentes áreas^(27)^.

Os depoimentos evidenciam a necessidade da capacitação contínua dos professores para que possam acompanhar as atualizações dos sistemas digitais e integrá-los à formação. Isso porque, embora muitos docentes não apresentem dificuldades com informática, a falta de treinamento específico no uso do PEP pode constituir um obstáculo relevante para a prática pedagógica, como evidenciado nos relatos de estudantes e docentes. Assim, a formação docente precisa considerar o letramento digital, indo além do simples uso de ferramentas, a fim de promover uma prática pedagógica crítica, colaborativa e contextualizada ^(13)^.

A fragmentação curricular e a abordagem tardia do PEP foram apontadas tanto por estudantes quanto por professores como fatores que dificultam o aprendizado e a aplicação do sistema. Esse cenário nos mostra que o avanço da transformação digital na saúde exige o desenvolvimento de competências profissionais alinhadas à conectividade e ao trabalho em rede, o que pressupõe uma formação contínua e adaptada à realidade tecnológica das instituições ^(13)^.

Os resultados elucidam que o letramento digital ainda representa um desafio para parte dos estudantes, especialmente aqueles com menor familiaridade com o uso de computadores. Esse achado dialoga com estudo que compreende o letramento digital para além do domínio técnico, incluindo a capacidade de interpretar, compreender e produzir informações ^(10)^. No presente estudo, as dificuldades observadas no uso do PEP indicam que as habilidades relacionadas às tecnologias digitais nem sempre são desenvolvidas linearmente no processo formativo, o que reforça a necessidade de estratégias pedagógicas inovadoras que promovam a apropriação crítica e significativa dessas tecnologias na formação técnica em enfermagem ^(28)^.

No campo docente, observou-se a necessidade de capacitação específica para o uso de ferramentas digitais. Embora muitos professores tenham experiência assistencial, ainda há lacunas no preparo pedagógico para trabalhar com sistemas digitais de saúde. Essa situação converge com resultados de uma pesquisa que mostrou a insuficiente preparação de docentes e estudantes para a saúde digital e o descompasso entre formação e prática profissional^(26)^. Docentes de cursos técnicos em enfermagem enfrentam desafios relacionados à diversidade de perfis estudantis, desinteresse e baixa preparação inicial dos alunos, de modo que essas dificuldades demandam reflexão crítica sobre práticas educativas e formação continuada docente ^(29)^. Somado a isso, a falta de padronização dos sistemas utilizados nas instituições aumenta a complexidade do ensino, exigindo constante adaptação dos estudantes e dificultando a consolidação do conhecimento prático ^(26)^.

Sob outra perspectiva, há um reconhecimento coletivo sobre a relevância do PEP como ferramenta essencial para a segurança do paciente e para a integração das informações assistenciais. A Resolução COFEN nº 754/2024 dispõe sobre o uso do prontuário eletrônico do paciente e de plataformas digitais na prática da enfermagem no Brasil, estabelecendo diretrizes para o registro seguro, sigiloso e ético das informações assistenciais ^(7)^. Ela ressalta a responsabilidade do profissional de enfermagem ao utilizar registros eletrônicos como instrumentos de gestão e de qualificação da assistência, evidenciando a necessidade de desenvolver essas competências desde a formação inicial ^(1)^.

Dessa forma, as dificuldades tecnológicas no uso do prontuário eletrônico do paciente impactam o processo de trabalho da equipe técnica de enfermagem, indicando que a construção de competências digitais, alinhadas às exigências do mercado de trabalho, deve ser integrada de forma transversal na formação técnica, fortalecendo o letramento digital, a criticidade e a autonomia profissional ^(30)^. Os resultados indicam, portanto, a importância de revisar o PPP, inserindo práticas pedagógicas interdisciplinares que promovam o desenvolvimento de competências digitais dos estudantes, atividades de simulação realística enquanto estratégia de ensino e ações de capacitação docente continuada, de modo que a formação acompanhe as demandas do cenário digital da saúde contemporânea ^(31)^.

### Limitações do estudo

Este estudo apresenta limitações metodológicas. Foi realizado em uma única instituição; além disso, utilizou uma abordagem qualitativa, o que não permite a generalização dos resultados para outras populações ou contextos.

### Contribuições para a área

Os resultados evidenciam lacunas na formação de técnicos de enfermagem no manejo de sistemas digitais como o PEP. Também destacam a importância da formação continuada de professores para aprimorar a prática pedagógica e integrar efetivamente as tecnologias digitais no ensino.

## CONSIDERAÇÕES FINAIS

Os resultados sugerem que o ensino técnico em Enfermagem apresenta avanços pontuais, porém ainda carece de consolidação no que se refere à inclusão sistemática de competências digitais relacionadas a ferramentas como o PEP. Observa-se que o desenvolvimento dessas competências permanece fragmentado, refletindo lacunas no planejamento pedagógico e na integração efetiva das tecnologias digitais aos componentes curriculares.

Entre as fragilidades identificadas, ressalta-se a introdução tardia do PEP no percurso formativo, a limitação de experiências simuladas que antecedem o contato com o sistema em campo de estágio e a insuficiente articulação entre teoria e prática. A diversidade de sistemas eletrônicos utilizados nas instituições de saúde e a ausência de formação complementar específica também se configuram como fatores que dificultam a apropriação do PEP pelos estudantes e docentes. Já as potencialidades observadas apontam para o reconhecimento, por parte dos docentes, da relevância do PEP como recurso pedagógico. Tal percepção constitui um ponto de partida favorável para fortalecer as práticas educativas ligadas ao uso de tecnologias digitais, desde que apoiadas por processos formativos consistentes.

Nesse sentido, as sugestões decorrentes dos achados incluem o aprimoramento do PPP, de modo que o ensino do PEP, alinhado às competências digitais, seja contemplado de forma transversal e contínua ao longo de todo o curso. A ampliação da carga horária destinada às disciplinas voltadas à informática e às tecnologias em saúde, a implementação de simuladores eletrônicos e a oferta de oficinas e atividades complementares também são medidas que podem favorecer a consolidação do letramento digital.

Salienta-se, ainda, a importância da formação continuada dos docentes, reconhecendo que o desenvolvimento da competência pedagógica é um processo permanente, sustentado tanto pela atualização técnica quanto pela reflexão crítica sobre a própria prática. O fortalecimento do letramento digital no ensino técnico em Enfermagem depende de uma articulação entre políticas institucionais, práticas pedagógicas e investimento na qualificação docente, de modo que a formação profissional acompanhe as transformações tecnológicas e responda às exigências contemporâneas da atenção à saúde.

## Data Availability

Os dados de pesquisa só estão disponíveis mediante solicitação.
